# Association Between Upper Limb Injury and Risk of Falls: A Nationwide Population-Based Cohort Study

**DOI:** 10.3390/jcm15135002

**Published:** 2026-06-26

**Authors:** Jhen-Jhen Fan, Wen Chi Chan, Jen-Hung Wang, Pao Huang, Ching-I Hong, Kuang-Ting Yeh

**Affiliations:** 1 School of Medicine, Tzu Chi University, Hualien 970374, Taiwan; 111311107@gms.tcu.edu.tw (J.-J.F.); 111311105@gms.tcu.edu.tw (W.C.C.); 2 Department of Medical Research, Hualien Tzu Chi Hospital, Buddhist Tzu Chi Medical Foundation, Hualien 970473, Taiwan; paulwang@tzuchi.com.tw; 3 Department of Special Education, National Dong Hwa University, Hualien 974301, Taiwan; 4 Department of Orthopedics, Hualien Tzu Chi Hospital, Buddhist Tzu Chi Medical Foundation, Hualien 970473, Taiwan; 5 Institute of Medical Sciences, Tzu Chi University, Hualien 970374, Taiwan; 6 Graduate Institute of Clinical Pharmacy, Tzu Chi University, Hualien 970374, Taiwan

**Keywords:** upper limb injury, falls, cohort study risk factors, older adults

## Abstract

**Background/Objectives:** Falls and upper limb injuries (ULI) are prevalent in older adults, yet whether ULI independently predisposes to subsequent falls remains poorly characterized. This nationwide cohort study evaluated the association between ULI and future fall risk using Taiwan’s National Health Insurance Research Database (2011–2019, follow-up through 2020). **Methods:** Adults aged ≥ 50 years with newly diagnosed ULI—defined as fractures (clavicle, scapula, humerus, radius, ulna, hand), sprains, strains, or open wounds of the shoulder, arm, elbow, forearm, wrist, or hand—were propensity score-matched 1:1 to controls by age, sex, and eight major comorbidities. Fall occurrence was identified by validated ICD codes, and Cox regression estimated hazard ratios (HRs) with 95% confidence intervals (CIs). **Results:** The cohort included 110,600 participants (mean follow-up 4.4 years). Fall incidence was 2.8 versus 1.6 per 1000 person-years in ULI versus control groups. Patients with ULI had 62% higher fall risk (adjusted HR 1.62, 95% CI: 1.43–1.84, *p* < 0.001), corresponding to 1.2 additional falls per 1000 person-years. Kaplan–Meier curves showed early divergence sustained throughout follow-up. **Conclusions:** ULI is independently associated with subsequent fall risk in older adults and may serve as a sentinel marker warranting fall-prevention strategies in clinical practice.

## 1. Introduction

Falls represent a major public health concern, particularly among older adults, with substantial implications for morbidity, mortality, and healthcare costs worldwide. The global burden of falls continues to increase as populations age, making fall prevention a critical priority for healthcare systems. In the context of aging populations, understanding the sequelae of upper limb injuries (ULI) extends beyond fracture healing; functional decline, fear of falling, and altered biomechanics following ULI may collectively increase vulnerability to future falls, creating a potentially self-reinforcing cycle of injury and disability in older adults. Identifying ULI as a sentinel marker for fall risk would have direct implications for geriatric assessment protocols and multidisciplinary rehabilitation planning. Although numerous risk factors for falls have been identified, including advanced age, medication use, cognitive impairment, and environmental hazards, the relationship between ULI and the risk of subsequent falls remains inadequately explored in large-scale epidemiological studies.

Recent literature suggests a complex relationship between upper limb function and falls risk [1,2,3,4]. Upper limb injuries may increase fall risk through several interconnected pathophysiological mechanisms. Biomechanically, immobilization with casts or slings restricts arm swing, which is essential for maintaining gait symmetry and dynamic stability during walking [5,6]. Neurologically, injury to joints and soft tissues impairs proprioceptive feedback from mechanoreceptors in the shoulder, elbow, wrist, and hand, reducing the body’s ability to sense limb position and respond rapidly to postural perturbations [7,8]. Functionally, loss of upper limb strength and coordination diminishes protective brace responses—the rapid arm movements used to break a fall or regain balance during unexpected trips or slips [4,9]. Psychologically, acute pain and fear of reinjury can lead to activity restriction and reduced mobility confidence, paradoxically increasing vulnerability to falls through muscle deconditioning and kinesiophobia [10,11]. Finally, upper limb injuries—particularly fragility fractures such as distal radius and proximal humerus fractures—may serve as sentinel markers of underlying frailty, osteoporosis, and sarcopenia, systemic conditions that independently predispose older adults to falls [12,13,14].

Studies have demonstrated that impaired upper limb motor function, altered protective arm reactions, and reduced dynamic stability following injury may contribute to increased vulnerability to falls. The study by Sultanoğlu et al. emphasizes that upper limb motor function can be a significant determinant of falls [1]. They observed significant changes in upper limb function assessments, and although improved upper limb function is expected to reduce fall risk, their study found no significant differences in fall rates between groups, suggesting that the relationship may be complex and influenced by factors such as overall mobility and strength [1]. The impact of upper limb impairment on balance and mobility has been demonstrated in specific populations. Major studies reported that individuals with upper limb loss exhibit a higher likelihood of falling due to altered motor responses and challenges in balance when adapting to prosthetic use [2]. This highlights the need for targeted interventions to improve balance and protective responses during falls, particularly in populations with ULI or prosthetic devices [2]. Similarly, Barbareschi et al. reported that poor transfer performance in wheelchair users increases the risk of falls and associated ULI [3]. The protective function of the upper limbs during falls has been extensively studied, revealing important insights into injury mechanisms. Komisar et al. examined mechanisms of fall-related injuries among older adults, emphasizing the importance of upper limb protective responses in reducing injury severity [4]. The high incidence of ULI during falls supports the need for proactive measures to mitigate fall risks, particularly in elderly populations [4]. Comorbid conditions further complicate this relationship. Diabetes is a risk factor for both ULI and falls, as it can cause complications affecting balance and mobility [15]. Akan reported that conditions predisposing older adults to falls also contribute to upper extremity injuries, including common distal radius fractures [12]. Furthermore, Yeung et al. found that sarcopenia, characterized by declines in muscle mass and strength, plays an important role in increasing fall risk and associated injuries, including those of the upper limbs [13].

Despite these findings, several critical gaps remain. Most studies have focused on specific populations or examined how falls result in ULI, yet few have investigated whether having a ULI independently increases the risk of subsequent falls in the general population. We focused on adults aged ≥ 50 years, as this population experiences the highest burden of both fragility-related upper limb injuries and fall-related morbidity, and represents the target demographic for fall prevention interventions in clinical practice [16,17]. We hypothesized that upper limb injury independently increases the risk of subsequent falls in adults aged ≥ 50 years, through mechanisms including impaired proprioception, reduced protective arm reactions, compensatory gait alterations, and psychological sequelae such as fear of reinjury. Therefore, this study aims to investigate the association between ULI and the risk of subsequent falls using a nationwide population-based cohort design. Using Taiwan’s National Health Insurance Research Database, we sought to determine whether individuals aged ≥ 50 years with ULI had a higher risk of falls compared with propensity score-matched controls, while controlling for potential confounding factors, including demographics and comorbidities. The findings will provide essential evidence for fall prevention strategies and inform clinical decision-making on risk assessment for patients with ULI.

## 2. Materials and Methods

### 2.1. Study Design and Data Source

We conducted a retrospective population-based cohort study leveraging Taiwan’s National Health Insurance Research Database (NHIRD), a comprehensive administrative claims registry encompassing virtually the entire Taiwanese population of approximately 23 million individuals. This database systematically archives longitudinal records of outpatient visits, inpatient admissions, surgical procedures, pharmaceutical dispensing, and patient demographics, thereby offering a robust infrastructure for nationwide epidemiological inquiry. This study was conducted in accordance with the Declaration of Helsinki and was approved by the Research Ethics Committee of Hualien Tzu Chi Hospital, Buddhist Tzu Chi Medical Foundation (REC No.: IRB112-133-C; approval date: 9 June 2023). Informed consent was waived given the retrospective nature of the study and the de-identified format of the NHIRD data.

### 2.2. Study Population and Matching Process

**Figure 1:**
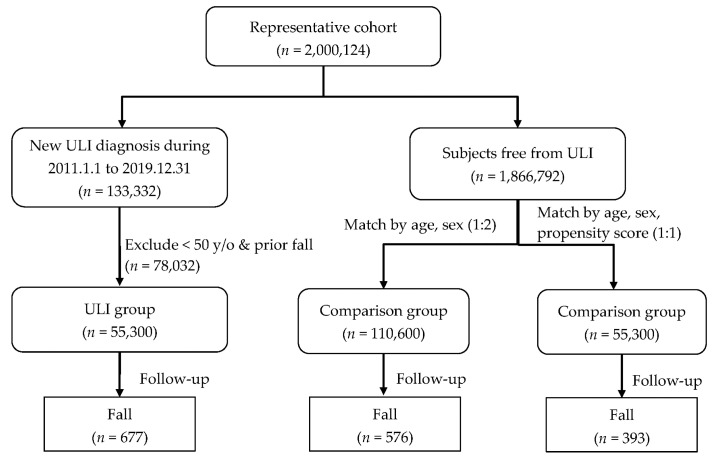
Study participant selection and matching flowchart.

Figure 1 illustrates the study cohort derivation process and the approach to patient selection and matching. From an initial representative cohort of 2,000,124 individuals in the NHIRD, we identified 133,332 patients with newly diagnosed ULI during the study period from 2011 to 2019. To ensure study validity and reduce confounding, we excluded patients aged < 50 years and those with a prior history of falls, resulting in 55,300 patients with ULI eligible for analysis. The age restriction was implemented because fall risk increases markedly after age 50, and including younger patients might dilute the association between ULI and falls. Prior fall history was defined by documented fall diagnoses using ICD-9-CM codes E880–E888 and ICD-10-CM codes W00–W19 recorded before the index date; excluding these patients helped establish a clear temporal relationship between ULI exposure and subsequent fall events.

We acknowledge that distal radius and proximal humerus fractures are frequently classified as fragility fractures and may, in some cases, result from falls, which could introduce circularity into the study design. To mitigate this concern, the exclusion of patients with any coded fall diagnosis prior to the index date was applied consistently across both groups, ensuring that the study captures new-onset fall events occurring after ULI rather than recurrent falls in individuals with pre-existing fall vulnerability. Nevertheless, we recognize that administrative exclusion based on coded diagnoses may not capture all prior fall events, particularly those that did not result in medical consultation, and this represents an inherent limitation of claims-based research.

For the comparison group, we initially selected 1,866,792 individuals without a ULI diagnosis during the study period. From this population, 110,600 potential controls were identified after applying the same exclusion criteria (age ≥ 50 years and no prior fall history). To enhance group comparability and mitigate selection bias, a two-stage matching procedure was implemented. In the first stage, candidate controls were pre-selected by matching on age and sex at a 2:1 ratio. In the second stage, propensity scores were derived from a logistic regression model incorporating age, sex, and all predefined baseline comorbidities, and 1:1 nearest-neighbor matching without replacement was applied. This sequential approach ultimately yielded 55,300 matched case–control pairs. Post-matching balance was confirmed by standardized mean differences (SMD) below 0.1 for all covariates, indicating adequate comparability across the two groups.

### 2.3. Exposure Definition and Diagnostic Criteria

Upper limb injury was defined using both ICD-9-CM and ICD-10-CM diagnostic codes to capture the transition period in Taiwan’s healthcare coding system. The ICD-9-CM codes included fractures of the upper limb (810–819), covering the clavicle, scapula, humerus, radius, ulna, and bones of the hand. Additionally, we included sprains and strains (840–842) and open wounds (880–887) of the upper limb, encompassing the shoulder, upper arm, elbow, forearm, wrist, and hand. For the ICD-10-CM system, we used codes S40–S49, which represent injuries to the shoulder and upper arm, including fractures, dislocations, sprains, strains, and soft tissue injuries.

To enhance the specificity of ULI ascertainment and minimize misclassification arising from administrative coding errors, a validated diagnostic confirmation algorithm was applied: patients were required to have either a minimum of two outpatient encounters carrying a ULI diagnosis code, or at least one hospitalization in which ULI was recorded as a primary or secondary discharge diagnosis. This confirmation strategy has been widely adopted in prior NHIRD-based research and has demonstrated acceptable validity for identifying true clinical cases within claims data. Patients had to be aged ≥ 50 years at the time of ULI diagnosis, as this age group represents the population at highest risk for upper limb injuries and subsequent falls due to age-related changes in bone density, muscle strength, and balance.

### 2.4. Comorbidity Assessment

Preexisting comorbid conditions were ascertained from inpatient and outpatient diagnostic records using both ICD-9-CM and ICD-10-CM coding systems, applying case-identification algorithms that have been previously validated within the NHIRD research framework. The assessment covered eight major conditions associated with fall risk. Hypertension encompassing essential hypertension, hypertensive heart disease, hypertensive kidney disease, and secondary hypertension, was defined using ICD-9-CM codes 401–405 and ICD-10-CM codes I10-I13 and I15. Diabetes mellitus, including type 1, type 2, and other specified diabetes mellitus, was defined using ICD-9-CM code 250 and ICD-10-CM codes E10, E11, and E13. Hyperlipidemia, representing disorders of lipoprotein metabolism and other dyslipidemias, was defined using ICD-9-CM code 272 and ICD-10-CM code E78. Coronary artery disease, including acute myocardial infarction, unstable angina, and chronic ischemic heart disease, was defined using ICD-9-CM codes 410–414 and ICD-10-CM codes I20-I22, I24, and I25. Cerebrovascular accidents, encompassing hemorrhagic stroke, ischemic stroke, and transient ischemic attacks, were defined using ICD-9-CM codes 430–438 and ICD-10-CM codes I60–I69, G45, and G46. Chronic liver disease, including alcoholic liver disease, chronic hepatitis, fibrosis, and cirrhosis of the liver, and other chronic forms, was defined using ICD-9-CM code 571 and ICD-10-CM codes K70, K73, K74, K754, K760, K769, K7581, and K7689. Chronic renal failure, representing chronic kidney disease stages 4–5 and unspecified chronic kidney disease, was defined using ICD-9-CM code 585 and ICD-10-CM codes N184–N186 and N189. Depression, including major depressive disorder, anxiety disorders, and adjustment disorders, was defined using ICD-9-CM codes 311, 2962, 2963, and 3004, and ICD-10-CM codes F30, F32, F40, F41, F43, F44, and F68.

### 2.5. Outcome Definition and Follow-Up

The primary outcome of interest was the occurrence of falls during the follow-up period. Falls were defined using specific ICD diagnostic codes for both accidental falls and documented fall history. Accidental falls, which include falls on the same level, falls from height, falls involving stairs, ladders, or other structures, were identified using ICD-9-CM codes E880–E888 and ICD-10-CM codes W00–W19. Additionally, we included codes for falls and repeated falls using ICD-9-CM codes 781.99 (other symptoms involving nervous and musculoskeletal systems) together with V15.5, V15.6, V15.89, and V15.9 (personal history of injury), and ICD-10-CM codes R29.6 (repeated falls) and Z91.8 (Z91.81, Z91.82, Z91.89) representing personal history of falls. It is important to note that the “history of falls” codes (ICD-9: V15.5–V15.9; ICD-10: Z91.81, Z91.82, Z91.89) refer to falls occurring after the index date that were documented during subsequent clinical encounters, not falls occurring before study entry, which were an exclusion criterion. Patients were followed from the index date (date of ULI diagnosis for the exposed group or matched date for controls) until the occurrence of the first fall event, death, withdrawal from the National Health Insurance program, or the end of the study period (31 December 2020), whichever occurred first. This approach facilitated comprehensive outcome ascertainment while accounting for competing risks and loss to follow-up.

### 2.6. Statistical Analysis

Between-group differences in baseline characteristics were evaluated using Pearson’s chi-square test for categorical variables and the independent-samples t-test for continuous variables. The adequacy of propensity score matching was assessed by computing SMDs for each covariate, with a threshold of SMD < 0.1 considered indicative of satisfactory balance. Fall incidence was expressed as event counts per 1000 person-years of observation. The association between ULI and fall risk was quantified using Cox proportional hazards regression, yielding HRs and corresponding 95% CIs from both unadjusted and fully adjusted models. The latter incorporated age, sex, and all eight comorbidities listed in Table 1. Although propensity score matching successfully balanced measured confounders between groups, we additionally adjusted for these variables in the Cox regression model to account for any residual imbalance and to provide doubly robust estimates. This approach is consistent with recent methodological recommendations for enhancing the precision of treatment effect estimates in matched cohort studies.

We used standard Cox proportional hazards regression to estimate the cause-specific hazard of falls. Although competing risk methods (e.g., Fine–Gray subdistribution hazard model) could provide additional insights into the subdistribution hazard in the presence of mortality as a competing event, our primary objective was to quantify the independent association between ULI and fall risk among those still alive and at risk, for which cause-specific hazards are appropriate and directly interpretable. The cause-specific hazard ratio represents the instantaneous rate of falls in the ULI group relative to controls among individuals who have not yet experienced a fall or died, which aligns with our clinical research question.

Adherence to the proportional hazard’s assumption was evaluated through Schoenfeld residual analysis. All statistical analyses were performed using SAS software, version 9.4 (SAS Institute Inc., Cary, NC, USA). Statistical significance was defined as a two-tailed *p*-value < 0.05.

## 3. Results

### 3.1. Baseline Characteristics and Matching Effectiveness

After completion of the 1:1 propensity score matching procedure, the analytic cohort consisted of 110,600 individuals distributed equally between the ULI-exposed group and the matched control group (*n* = 55,300 each). Covariate balance was excellent across all measured baseline variables, as evidenced by SMDs uniformly below 0.1 (Table 1). Both groups had a comparable mean age of approximately 65 years (control: 64.66 ± 12.02 years; ULI: 64.97 ± 12.30 years), and male participants constituted approximately 59.5% of each group. The 50–65-year age stratum represented the largest subgroup in both cohorts (controls: 56.6%; ULI group: 56.0%). The distribution of all eight prespecified comorbidities—including hypertension, diabetes mellitus, hyperlipidemia, coronary artery disease, cerebrovascular accident, chronic liver disease, chronic renal failure, and depression—was virtually identical between groups, with SMDs ranging from 0.00 to −0.01. The only variable with a nominally higher SMD was fall history (SMD = 0.05; 0.7% in controls vs. 1.2% in the ULI group, *p* < 0.001), which remained clinically negligible. The mean follow-up time was 4.49 ± 2.28 years in the control group and 4.43 ± 2.29 years in the ULI group, totaling 492,997 person-years of observation.

Regarding the distribution of ULI subtypes within the exposed group, fracture-related injuries—including fractures of the clavicle, scapula, humerus, radius, ulna, and hand bones (ICD-9-CM 810–819; ICD-10-CM S40–S49)—constituted the majority of cases, consistent with the epidemiological profile of fragility fractures in adults aged ≥ 50 years. Soft tissue injuries, including sprains, strains, and open wounds (ICD-9-CM 840–842, 880–887), represented a smaller proportion of the cohort. The heterogeneity of ULI types is acknowledged as a potential source of variability in the observed association with fall risk, and future studies with granular injury-type data are encouraged to examine differential effects across ULI subtypes.

### 3.2. Fall Incidence and Risk Assessment

The primary outcome analysis, detailed in Table 2, demonstrates a significant association between upper limb injury and subsequent fall risk. During follow-up, 677 falls occurred in the ULI group compared with 393 cases in the control group. The total person-years of follow-up were 244,832 in the ULI group and 248,165 in the control group. The crude incidence rate of falls was markedly higher in the ULI group (2.8 per 1000 person-years) than in the control group (1.6 per 1000 person-years), representing a 75% higher incidence rate. In the univariate Cox proportional hazard model, patients with ULI demonstrated a crude hazard ratio of 1.64 (95% CI: 1.45–1.86, *p* < 0.001). After comprehensive adjustment for age, sex, hypertension, diabetes mellitus, hyperlipidemia, coronary artery disease, cerebrovascular accident, chronic liver disease, chronic renal failure, and depression, the adjusted hazard ratio remained consistent at 1.62 (95% CI: 1.43–1.84, *p* < 0.001). The minimal attenuation between crude and adjusted estimates indicates that propensity score matching effectively controlled for measured baseline differences. Subgroup analyses stratified by age group and sex consistently demonstrated elevated fall risk in the ULI group across all strata (Table 3), supporting the robustness of the primary finding.

### 3.3. Survival Analysis and Time-to-Event Assessment

**Figure 2:**
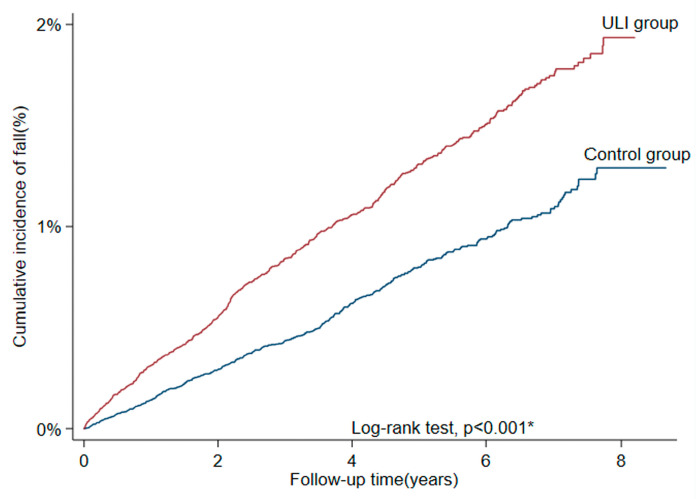
Kaplan–Meier survival curves for fall-free survival. * *p*-value < 0.05 was considered statistically significant after test.

Kaplan–Meier analysis demonstrated a clear and persistent separation in fall-free survival between the ULI and control groups throughout the entire follow-up period (Figure 2). Divergence of the survival curves became apparent within the first year following the index date and continued to widen over subsequent years, indicating that the elevated fall risk associated with ULI persists well beyond the acute injury phase. At 1, 2, 3, and 4 years of follow-up, the numbers at risk were approximately 52,000, 48,000, 43,000, and 38,000 in each group, respectively. The log-rank test confirmed statistically significant differences in fall-free survival between groups (*p* < 0.001). The sustained separation of the curves is consistent with the proportional hazards’ assumption, as verified by Schoenfeld residual analysis.

## 4. Discussion

This nationwide propensity-matched cohort study, including 110,600 participants (55,300 patients with ULI and 55,300 controls) with a mean follow-up of 4.4 years, found that individuals with ULI had a significantly higher risk of subsequent falls. The adjusted hazard ratio was 1.62 (95% CI: 1.43–1.84, *p* < 0.001), corresponding to a 62% increase in risk. The incidence rate of falls was 2.8 per 1000 person-years in the ULI group compared with 1.6 per 1000 person-years in controls. Kaplan–Meier survival analysis showed that fall-free survival curves diverged within the first year after injury and continued to separate throughout the four-year follow-up period. Subgroup analyses stratified by age group and sex consistently demonstrated elevated fall risk in the ULI group across all strata (Table 3), supporting the robustness of the primary finding. These findings are consistent with ULI serving as both an acute destabilizing event and a persistent risk factor for falls among middle-aged and older adults.

The early divergence of survival curves suggests that mechanisms operating in the acute phase after ULI are particularly important. First, immobilization with casts or slings restricts arm swing, alters gait symmetry, and reduces dynamic stability [5]. Second, pain and fear of reinjury can lead to activity restriction, decreasing mobility confidence, and increasing vulnerability to falls [6,7]. Third, pharmacological factors may also contribute to elevated fall risk following ULI. Opioids and sedatives are commonly prescribed after musculoskeletal injuries, and both drug classes have been associated with increased fall risk in observational studies [8,9,10]. Although medication use was not directly examined in the present study, this represents a plausible biological mechanism that warrants investigation in future research with access to prescription data. These acute-phase factors explain why the ULI cohort exhibited higher fall incidence soon after the index injury and highlight the critical need for early preventive strategies during recovery [11,14,16]. The persistence of elevated fall risk throughout the follow-up period suggests that long-term consequences also play a crucial role. Functionally, impaired upper limb function has been shown to limit protective arm reactions and balance recovery during unexpected perturbations, potentially compromising stability even after apparent clinical healing [17,18]. Biomechanically, incomplete recovery of range of motion, coordination, and strength may leave patients less able to stabilize themselves or protect against injury during a fall [18]. Systemically, ULI often represents a sentinel marker of underlying frailty rather than a direct causal factor. Distal radius and proximal humerus fractures, which constitute a substantial proportion of upper limb injuries in older adults, are recognized as fragility fractures strongly associated with osteoporosis and sarcopenia [19,20]. These conditions predict recurrent falls and fractures in large population studies [13,21,22]. The sustained separation of survival curves observed in our study likely reflects not only short-term biomechanical destabilization but also the unmasking of pre-existing musculoskeletal vulnerability that predisposes to future fall events. In this context, ULI may function as a “canary in the coal mine”—an early clinical indicator of systemic frailty that warrants comprehensive geriatric assessment and multifactorial intervention [23,24].

From an absolute risk perspective, the incidence of falls was 2.8 per 1000 person-years in the ULI group compared with 1.6 per 1000 person-years in controls, corresponding to an absolute risk difference of 1.2 additional falls per 1000 person-years. Over the mean follow-up of 4.4 years, this translates to approximately 5.3 additional falls per 1000 individuals with ULI. While the absolute incidence remains relatively low, the high prevalence of ULI in older adults (55,300 cases identified over 9 years in our cohort) means that even modest absolute risk increases have substantial public health implications. Furthermore, the 62% relative risk increase (aHR 1.62) is clinically meaningful and comparable to established fall risk factors such as polypharmacy and gait impairment [24,25]. Given that falls are the leading cause of injury-related morbidity and mortality in older adults, with associated healthcare costs exceeding billions of dollars annually, interventions targeting even small absolute risk reductions in high-prevalence conditions like ULI could yield significant population-level benefits [26].

Most previous studies have investigated how falls result in ULI—particularly fractures of the wrist and shoulder. In contrast, our study examined the reverse relationship, whether the occurrence of ULI increases the risk of subsequent falls [27,28]. By demonstrating a significant bidirectional association, our results provide novel evidence that ULI is not merely a consequence of falls but also an independent predictor of future falls. This finding fills an important gap in the current understanding of fall risk factors and establishes ULI as a clinically relevant marker for fall prevention interventions. Prior research has also focused heavily on postmenopausal women. In this group, estrogen deficiency, osteoporosis, and low-energy wrist fractures are viewed as early indicators of recurrent injury [20,21,22,29]. Regarding sex-specific fall risk, our subgroup analysis revealed that the association between ULI and subsequent falls was statistically significant in both males (adjusted HR 1.59, 95% CI: 1.35–1.87) and females (adjusted HR 1.67, 95% CI: 1.37–2.05), with a modestly higher point estimate observed in women. This finding is consistent with the widely recognized higher fall risk among older women, which has been attributed to greater prevalence of osteoporosis, lower muscle mass, and hormonal changes following menopause [23,30]. However, the magnitude of the ULI–fall association in men was nearly equivalent, suggesting that ULI confers substantial fall vulnerability regardless of sex. Prior research has predominantly focused on postmenopausal women, in whom estrogen deficiency, osteoporosis, and low-energy wrist fractures are viewed as early indicators of recurrent injury. Our sex-inclusive cohort extends these findings to male patients, thereby broadening the clinical relevance of ULI as a fall-risk marker beyond postmenopausal populations and supporting the implementation of fall-prevention strategies for all older adults sustaining ULI.

Our findings carry significant clinical implications for both orthopedic and geriatric practice. By showing that ULI is independently associated with a 62% higher fall risk, this study supports viewing ULI as a potential sentinel marker of broader fall vulnerability that may warrant systematic prevention efforts. We therefore propose that fall-risk screening should be initiated at the point of ULI diagnosis, rather than deferred to later stages of recovery. Validated screening tools—such as the STEADI (Stopping Elderly Accidents, Deaths & Injuries) algorithm, the Timed Up and Go test, or the FRAX fracture risk assessment—may be readily integrated into routine orthopedic consultations to identify patients requiring targeted intervention. Rehabilitation approaches should extend beyond restoring upper limb function alone. Evidence from large-scale fall-prevention trials demonstrates that exercise programs that incorporate lower-limb strengthening, gait training, and balance retraining reduce fall risk in community-dwelling adults, and such elements are likely equally important in patients recovering from ULI [11,14]. Specifically, multicomponent programs combining proprioceptive training, Tai Chi, and progressive resistance exercise have demonstrated efficacy in reducing fall rates by 20–30% in community-dwelling older adults and represent a practical model adaptable to the ULI rehabilitation context [14,28]. During the acute phase, patient education on safe mobility and home hazard modification is critical, particularly during immobilization, while comprehensive medication review should address fall-risk-increasing drugs, particularly sedatives and opioids [11,12]. Structured deprescribing protocols and pharmacist-led medication reconciliation at the time of injury represent actionable strategies that can be incorporated into existing orthopedic care pathways [11,31]. For long-term management, integrating multifactorial fall-risk assessment—including medication use, gait evaluation, environmental hazards, and comorbidity management—into orthopedic and rehabilitation services provides a practical framework for routine care. This approach has been shown to improve identification of high-risk individuals [14,16]. Patients identified as high-risk should be referred to dedicated falls clinics or geriatric assessment services, where interdisciplinary teams can deliver coordinated interventions addressing the full spectrum of fall determinants. At the healthcare system level, embedding fall-prevention protocols within orthopedic care pathways for ULI patients—analogous to existing post-fracture liaison service models—could translate the findings of this study into measurable reductions in fall-related morbidity and associated healthcare costs [32,33].

Despite the strengths of this study, several limitations inherent to its design must be acknowledged. First, falls were identified through administrative claims data, which capture only medically attended events. Minor stumbles and near-falls that did not result in medical consultation were not captured, likely leading to underestimation of true fall incidence [34,35]. Second, surveillance bias is a concern. Patients with ULI typically receive more intensive follow-up care than matched controls, which may increase the likelihood of fall detection in the ULI group and potentially inflate the observed association [35,36]. Third, the present study was unable to distinguish between high-energy and low-energy mechanisms of upper limb injury. Although our cohort was restricted to adults aged ≥ 50 years—a population in whom low-energy fragility injuries predominate—we cannot exclude the possibility that some injuries resulted from high-energy trauma, which may involve different pathophysiological pathways and fall-risk profile. Fourth, neurological conditions known to predispose individuals to falls, such as dementia and Parkinson’s disease, were not included as covariates in the propensity score model, as these diagnoses were not consistently captured in the NHIRD during the study period. Similarly, direct measures of frailty, osteoporosis severity, sarcopenia, medication use (particularly fall-risk-increasing drugs such as opioids, sedatives, and antihypertensives), and baseline functional status were unavailable. Residual confounding from these unmeasured variables cannot be excluded, and this represents an important limitation of the current analysis. Fifth, while falls were excluded based on ICD-coded diagnoses, patients without a recorded fall diagnosis may still have experienced unreported falls prior to study entry. The administrative exclusion criterion therefore reflects medically documented fall history rather than true fall-naïve status, and some degree of misclassification cannot be ruled out. Sixth, important clinical details—including injury severity, hand dominance, immobilization duration, rehabilitation adherence, and medication use (particularly opioids and sedatives)—were unavailable from the database. These factors are known to influence functional recovery and fall risk, and their absence limits the mechanistic interpretation of our findings [37,38,39]. Seventh, the broad definition of ULI in this study encompasses a heterogeneous spectrum of injuries, including fractures, sprains, open wounds, and soft tissue injuries. These conditions differ substantially in clinical severity, immobilization requirements, and functional impact. It is plausible that fragility fractures (e.g., distal radius, proximal humerus) confer higher fall risk than minor soft tissue injuries due to their association with underlying osteoporosis and frailty. Future studies with access to detailed clinical data should stratify analyses by injury type, mechanism (high-energy vs. low-energy), and treatment modality (surgical vs. conservative) to refine risk prediction models [19,39]. Eighth, while propensity score matching successfully balanced measured comorbidities, residual confounding from unmeasured variables—such as cognitive status, baseline physical activity, frailty severity, and environmental hazards—cannot be excluded [36,40]. Ninth, our cohort was restricted to adults aged ≥ 50 years, and caution is required when extrapolating these findings to younger populations, in whom upper limb injuries more commonly result from high-energy mechanisms and may carry different implications for fall risk [41]. Tenth, we were unable to perform sensitivity analyses using alternative analytical approaches (e.g., inverse probability of treatment weighting, different matching algorithms, or stratification by injury mechanism) due to expiration of data access. Future studies with ongoing database access should validate our findings using multiple analytical frameworks [42,43]. Finally, the observational design of this study precludes definitive causal inference. While our findings demonstrate a robust statistical association between ULI and subsequent falls after comprehensive adjustment for measured confounders, prospective intervention studies are required to confirm whether targeted fall-prevention programs initiated at the time of ULI can effectively reduce subsequent fall incidence in clinical practice.

Future research should investigate specific injury characteristics in greater detail to refine risk stratification and intervention strategies. Laterality may be particularly relevant, as fractures of the dominant extremity have been associated with greater functional disability and slower recovery, suggesting that hand dominance could markedly influence subsequent fall risk [37,38]. Similarly, injury severity and multiplicity warrant investigation, as multiple or bilateral upper limb injuries impose greater functional limitations and dependence in daily activities, which may translate into a higher risk of subsequent falls [39,41]. Prospective studies incorporating detailed clinical assessments, objective functional testing, and time-varying risk factors—including dynamic medication exposure and competing risks analysis—could clarify temporal dynamics and improve the development of individualized rehabilitation strategies [26,42]. Additionally, intervention studies testing the effectiveness of integrated fall prevention programs in patients with ULI would provide crucial evidence for clinical practice guidelines and healthcare policy development.

## 5. Conclusions

This nationwide cohort study with a mean follow-up of 4.4 years suggests that upper limb injury is independently associated with an approximately 62% increased fall risk in adults aged ≥ 50 years, with elevated risk persisting throughout follow-up. ULI may serve as a sentinel marker of fall vulnerability, warranting consideration of fall-prevention strategies in routine clinical care. A multifactorial approach encompassing comprehensive rehabilitation, medication review, and fall risk assessment may help reduce fall-related morbidity in this high-risk population. 

## Figures and Tables

**Table 1:** Baseline characteristics and comorbidity (*n* = 110,600).

Variables	Control (*n* = 55,300)	ULI (*n* = 55,300)	*p*-Value	SMD
Age (y/o)	64.66 ± 12.02	64.97 ± 12.30	<0.001 *	0.030
Age Group			<0.001 *	0.030
50–65 y/o	31,319 (56.6%)	30,955 (56.0%)		
65–80 y/o	15,000 (27.1%)	14,758 (26.7%)		
≥80 y/o	8981 (16.2%)	9587 (17.3%)		
Sex			0.883	0.000
Male	32,938 (59.6%)	32,914 (59.5%)		
Female	22,362 (40.4%)	22,386 (40.5%)		
Comorbidity				
HTN	22,276 (40.3%)	22,060 (39.9%)	0.185	−0.010
DM	10,945 (19.8%)	10,792 (19.5%)	0.247	−0.010
Hyperlipidemia	11,875 (21.5%)	11,752 (21.3%)	0.367	−0.010
CAD	6360 (11.5%)	6399 (11.6%)	0.714	0.000
CVA	4083 (7.4%)	4134 (7.5%)	0.559	0.000
Chronic liver disease	3367 (6.1%)	3307 (6.0%)	0.449	0.000
Chronic renal failure	1885 (3.4%)	1920 (3.5%)	0.564	0.000
Depression	2767 (5.0%)	2784 (5.0%)	0.815	0.000
Fall	393 (0.7%)	677 (1.2%)	<0.001 *	0.050
Follow-up time (year)	4.49 ± 2.28	4.43 ± 2.29	<0.001 *	−0.030

Data are presented as n and percentage. * *p*-value < 0.05 was considered statistically significant after test. Note: Although statistically significant differences in mean age (64.66 vs. 64.97 years, *p* = 0.020) and follow-up duration (4.49 vs. 4.43 years, *p* = 0.010) were observed due to the large sample size, the standardized mean differences (SMD < 0.03) indicate these differences are clinically negligible and do not compromise the validity of the propensity score matching. ULI, upper limb injuries; SMD, standardized mean difference; HTN, hypertension; DM, diabetes mellitus; CAD, coronary artery disease; CVA, cerebrovascular accident.

**Table 2:** Risk of fall in patients with and without ULI (*n* = 110,600).

Variables	ULI	
	Yes	No
Patient numbers	55,300	55,300
Fall cases	677	393
Person-years	244,832	248,165
Incidence rate ^a^	2.8	1.6
Univariate model		
crude HR (95% CI)	1.64 (1.45–1.86)	1 (ref.)
*p*-value	<0.001 *	
Multivariate model ^b^		
aHR (95% CI)	1.62 (1.43–1.84)	1 (ref.)
*p*-value	<0.001 *	

^a^ Per 1000 person-years. ^b^ Multivariate Cox proportional hazard regression model with adjustment for all baseline characteristics shown in Table 1. ULI, upper limb injuries; HR, hazard ratio; aHR, adjusted hazard ratio; CI, confidence interval; ref., reference. * *p*-value < 0.05 was considered statistically significant after test.

**Table 3:** Subgroup analysis of Cox’s regression model for the association between ULI and fall (*n* = 110,600).

Variables	Crude HR (95% CI)	*p*-Value	Adjusted HR (95% CI)	*p*-Value
**Main model**	1.00		1.00	
Control	1.64 (1.45–1.86)	<0.001 *	1.62 (1.43–1.84)	<0.001 *
ULI				
**Age**				
50–65 y/o	1.00		1.00	
Control	1.74 (1.41–2.14)	<0.001 *	1.76 (1.43–2.17)	<0.001 *
ULI				
65–80 y/o	1.00		1.00	
Control	1.52 (1.21–1.90)	<0.001 *	1.52 (1.21–1.90)	<0.001 *
ULI				
≥80 y/o	1.00		1.00	
Control	1.65 (1.32–2.06)	<0.001 *	1.62 (1.30–2.02)	<0.001 *
ULI				
**Sex**				
Male	1.00		1.00	
Control	1.60 (1.36–1.88)	<0.001 *	1.59 (1.35–1.87)	<0.001 *
ULI				
Female	1.00		1.00	
Control	1.70 (1.39–2.07)	<0.001 *	1.67 (1.37–2.05)	<0.001 *
ULI	1.00		1.00	

In all subgroup analyses, the Control group serves as the reference category (HR = 1.00), and hazard ratios are presented for the ULI group relative to controls within each stratum in the Cox’s proportional hazards model. Adjusted models control for age, sex, hypertension, diabetes mellitus, hyperlipidemia, coronary artery disease, cerebrovascular accident, chronic liver disease, chronic renal failure, and depression. ULI, upper limb injuries; HR, Hazard Ratio; CI, Confidence Interval. * *p*-value < 0.05 was considered statistically significant after test.

## Data Availability

The datasets generated and analyzed during the current study are not publicly available due to the restrictions of the Taiwan National Health Insurance Research Database (NHIRD) regulations and patient privacy protection requirements. Access to NHIRD data requires approval from the Taiwan Ministry of Health and Welfare and adherence to strict data security protocols. Researchers interested in accessing similar datasets may apply through the Health and Welfare Data Science Center, Ministry of Health and Welfare, Taiwan (https://dep.mohw.gov.tw/DOS/np-2497-113.html, accessed on 15 October 2023). The statistical code used for data analysis is available from the corresponding author upon reasonable request and with appropriate data use agreements in place.

## Abbreviations

The following abbreviations are used in this manuscript:

ULI	Upper limb injuries
HRs	Hazard ratios
CIs	Confidence intervals
NHIRD	National Health Insurance Research Database
SMD	Standardized mean differences
